# Organizers in a dish: Modeling human CNS morphogenesis

**DOI:** 10.1016/j.devcel.2026.01.003

**Published:** 2026-02-04

**Authors:** Georgina Miller, Daniel. J Lloyd-Davies Sánchez, José González Martínez, Alexander W. Justin, Madeline A. Lancaster, Luca Guglielmi

**Affiliations:** 1 MRC Laboratory of Molecular Biology, Cambridge, UK; 2 Department of Cellular, Computational and Integrative Biology (CIBIO), University of Trento, Trento, Italy

## Abstract

The human brain stands out for the scale of cellular and morphological complexity across anterior-posterior domains. Modeling the entire neuraxis is therefore essential to comprehend human neural development and disease. Brain organoids commonly recapitulate anterior regions due to the propensity of neural progenitors to acquire telencephalic identities and self-organize into cortical layers. In the embryo, posterior brain patterning is orchestrated by organizers, signaling centers positioned at anterior-posterior locations that are rarely induced *in vitro*. Several strategies have been developed to reproduce organizer signals, employing small molecules and recombinant morphogens, thereby expanding the *in vitro* repertoire of human neural identities. Despite this, posterior models do not yet reproduce the morphological complexity of their *in vivo* counterparts. In this review, we discuss how this discrepancy may stem from the inability to recapitulate the spatiotemporal dynamics of organizer activity and how recent technologies can balance guided differentiation and self-organization, enhancing the fidelity of human brain organoid models.

## Introduction

The complexity of the human brain emerges during embryogenesis, as the neural tube is patterned along anterior-posterior (A/P) and dorsal-ventral (D/V) axes into the telencephalon, diencephalon, mesencephalon, rhombencephalon, and spinal cord.^1,2^ While the cerebral cortex has drawn most attention for its evolutionary expansion and cognitive roles,^3,4^ other regions, including the hippocampus,^5^ thalamus,^6^ and cerebellum,^7–10^ also contribute to the overall increase in brain size.

Furthermore, many neurological disorders affect regions beyond the cortex, including Alzheimer’s disease, affecting the hippocampus,^11^ or cerebellar defects in ataxia telangiectasia.^12,13^ Notably, key aspects of these disorders cannot be fully replicated in other species, and additional perturbations are often required,^14,15^ suggesting that evolutionary differences in brain structure and function may, at least in part, explain disease susceptibility in humans.

**Figure 1:**
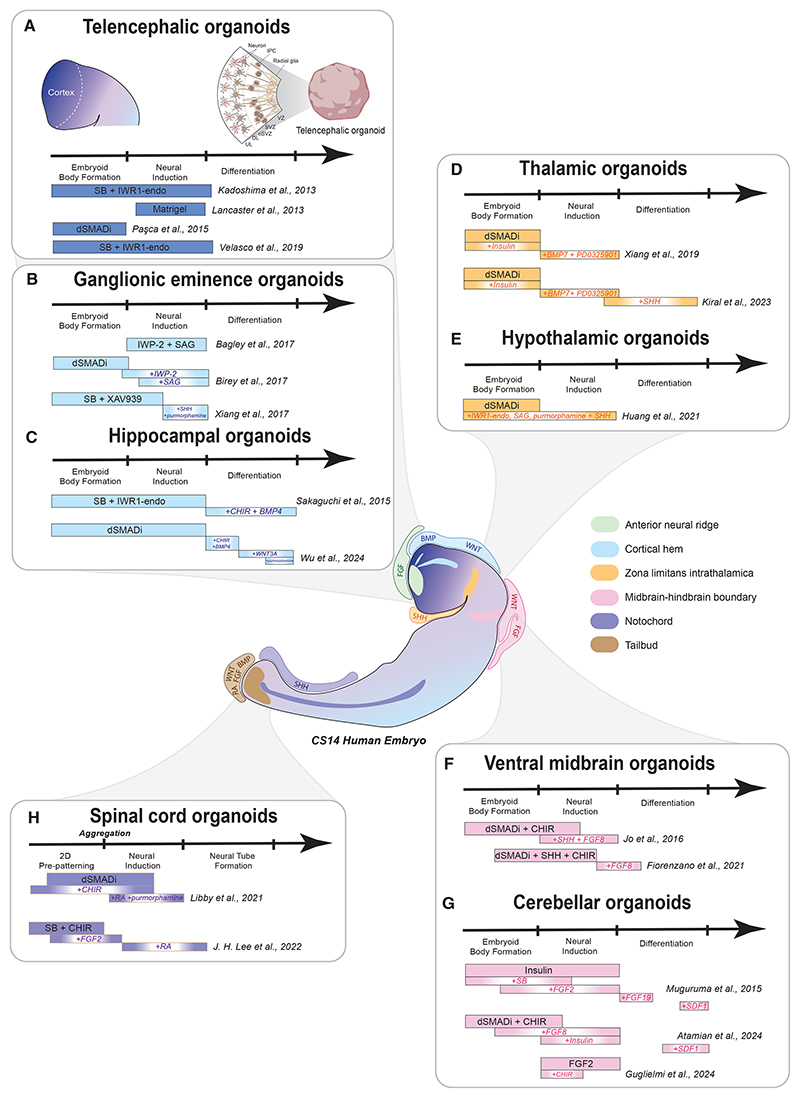
Regional brain organoids and related signaling centers (A) The telencephalon is a default state for neural progenitors. Early organoid protocols made use of this property to reproduce cell fate specification and morphogenesis of the cortex. IPCs, intermediate progenitor cells; UL, upper layer; DL, deep layer; oSVZ, outer subventricular zone; SVZ, subventricular zone; VZ, ventricular zone. (B) Ganglionic eminence organoids are generated from telencephalic organoids by activating SHH signaling to induce ventral identities. (C) Hippocampal development relies on WNT signaling from the hem. Accordingly, hippocampal organoids rely on WNT signaling activation upon establishment of telencephalic fate. (D) Thalamic organoids rely on slight posteriorization of telencephalic organoids using insulin; identities can be steered ventral and anterior through SHH activation. (E) Hypothalamic organoids necessitate even stronger SHH signaling activation. (F) Ventral midbrain organoids require reproducing FGF and WNT signaling from the MHB in addition to SHH. (G) Similar to (F), cerebellar organoids depend on FGF and WNT signaling activation, but not SHH. (H) Spinal cord organoids necessitate pre-patterning through WNT activation before aggregation to develop into posterior neural tissue.

Brain organoids offer a powerful tool to investigate the mechanisms underlying human neural development and disease.^16,17^ To date, most organoid models recapitulate the development of the telencephalon, the most anterior part of the brain that gives rise to the cerebral cortex.^18–21^ This modeling is facilitated by the relative ease in obtaining telencephalic identities. Indeed, anterior brain identities emerge as a default state for neural progenitors in the absence of caudalizing signals,^22^ a feature that is also maintained *in vitro*.^23^ Early organoid methods took advantage of this property by culturing human embryonic stem cells (hESCs) or induced pluripotent stem cells (hiPSCs) under “unguided” conditions (that is, without the addition of exogenous patterning molecules) and leveraging their self-organizing capacity^20,24,25^ (Figure 1A). In addition to specifying diverse cell fates, these models recapitulated tissue architecture, such as cortical layering, demonstrating that stem cells can undergo *in vivo*-like morphogenesis.^26,27^ Notably, under these conditions, the emergence of posterior brain identities is also occasionally observed. However, their proportions vary both quantitatively and qualitatively,^21^ limiting the reproducibility of such models for the study of the posterior brain.

Building on principles from classical developmental biology, the stem cell field has made significant advances in reproducing posterior brain regions. One particularly influential concept is that of the organizer, first described by Spemann and Mangold, as a signaling center capable of instructing cell fate specification and tissue morphogenesis.^28^ Classically, organizers were defined by their ability to impose regional identity upon xenotrans-plantation. An iconic example is the midbrain-hindbrain boundary (MHB), a key organizer that controls the development of the midbrain and cerebellum and can induce ectopic formation of these identities when grafted into other brain regions.^29^

Organizers exert their effects through the secretion of morphogens, signaling molecules that coordinate cell fate specification and morphogenesis through the establishment of signaling gradients.^30–33^ WNT, sonic hedgehog (SHH), fibroblast growth factor (FGF), bone morphogenetic protein (BMP), and retinoic acid (RA) signaling dynamically interact to instruct regional neural identities.^22,34–41^ Here, we review the strategies employed to mimic organizer signals *in vitro* through the application of recombinant ligands and small-molecule agonists or inhibitors and generate organoids resembling different regions of the human central nervous system (CNS). Beyond regionalized organoids, the field has begun to develop integrated systems, such as assembloids, synthetic neural tubes, and gastruloids, aimed at capturing the concerted emergence of neural identities and their interconnections across the developing CNS.

Coupled with the emergence of comprehensive single-cell RNA sequencing (scRNA-seq) datasets of the developing human brain, these approaches have shown that a broad repertoire of *in vivo* cell states can now be faithfully reproduced *in vitro*.^42,43^ Despite these advances, a substantial gap remains between these patterned models and *in vivo* morphogenesis. Indeed, posteriorization achieved through substantial ectopic guidance, typically via the uniform application of individual morphogen concentrations, often restricts differentiation to a subset of cell identities. While applying guidance in this form increases reproducibility, it often comes at the expense of neuronal diversity and the emergence of *in vivo* tissue-scale cytoarchitecture. We will discuss how combining organoid technologies with optogenetics, microfluidics, and synthetic organizers can help reproduce morphogen gradients displaying physiological ranges and improve modeling of human brain development.

## Patterning of Regional CNS Organoids

From anterior to posterior, the human CNS is patterned by a series of organizers. The anterior neural ridge (ANR)^44^ and the cortical hem (CH)^45^ pattern the telencephalon, whereas the zona limitans intrathalamica (ZLI) patterns the diencephalon.^46^ Further posteriorly, the MHB shapes the mesencephalon and rhomben-cephalon.^47^ Finally, the tailbud acts as an organizer-like center, driving spinal cord extension (Figures 1A–1H).^48^ Because posteriorizing morphogens, such as WNTs and FGFs, act broadly across germ layers, posterior organoid protocols typically suppress non-neural fates via dual SMAD inhibition (dSMADi) before inducing organizer-specific cues.^49^ Next, we review the major organizers of the human CNS, the strategies used to mimic their activity, and the remaining challenges in recapitulating their morphogenetic potential *in vitro*.

### ANR and CH

The telencephalon is subdivided into distinct regions: dorsally, the cerebral cortex; medially, the hippocampus and choroid plexus (CP); and ventrally, the basal ganglia, which include the lateral, caudal, and medial ganglionic eminences (LGE, CGE, and MGE, respectively).^34,50^ Renner et al. demonstrated that unguided brain organoids can spontaneously develop organizer centers *in vitro*, such as the CH, and reproduce specification of a range of D/V identities, such as cortex, GEs, and CP, within a continuous neuroepithelium.^26^ In the embryo, such spatial organization is normally driven by opposing rostrocaudal and medio-lateral FGF and WNT signaling gradients, respectively, from the ANR, located at the rostral-most tip of the telencephalic neuroepithelium,^51,52^ and the CH, positioned medially.^45,53^ These factors, in combination with SHH from the floor plate and BMPs from the roof plate, coordinate the specification of the full range of D/V telencephalic identities.^34,54^

Although unguided organoids can reproduce coordinated development of dorsal and ventral territories *in vitro*,^26^ their occurrence is variable, often favoring one fate over the other. Co-development of dorsal and ventral identities is critical, since the MGE and LGE generate many of the inhibitory interneurons of the cortex and hippocampus. These cells migrate tangentially to integrate into hippocampal and cortical circuits,^55^ a process essential for establishing balanced circuit activity.

More consistent specification of either dorsal or ventral identities has been achieved through dSMADi^19,56^ or combined WNT inhibition (IWR-1/Dkk-1) and transforming growth factor β (TGF-β) inhibition (SB-431542)^21,23,57^ (Figure 1A), albeit often at the expense of medial and ventral identities. Under such conditions, activation of the SHH signaling pathway directs patterning toward GE identity^56,58,59^ (Figure 1B) but tends to yield an enrichment of MGE-like progenitors with more limited representation of LGE or CGE territories.

Unlike dorsal and ventral identities, medial fates, such as the hippocampus, remain more challenging. *In vivo*, hippocampal development relies on synergistic WNT and BMP signaling from the CH.^53,60,61^ Wnt signaling is required for both cornu ammonis (CA) fields and dentate gyrus (DG) formation.^61,62^ Furthermore, sustained canonical Wnt signaling in the lateral cortex can induce cells with hippocampal characteristics, at the expense of cortical fate.^63^

**Figure 2:**
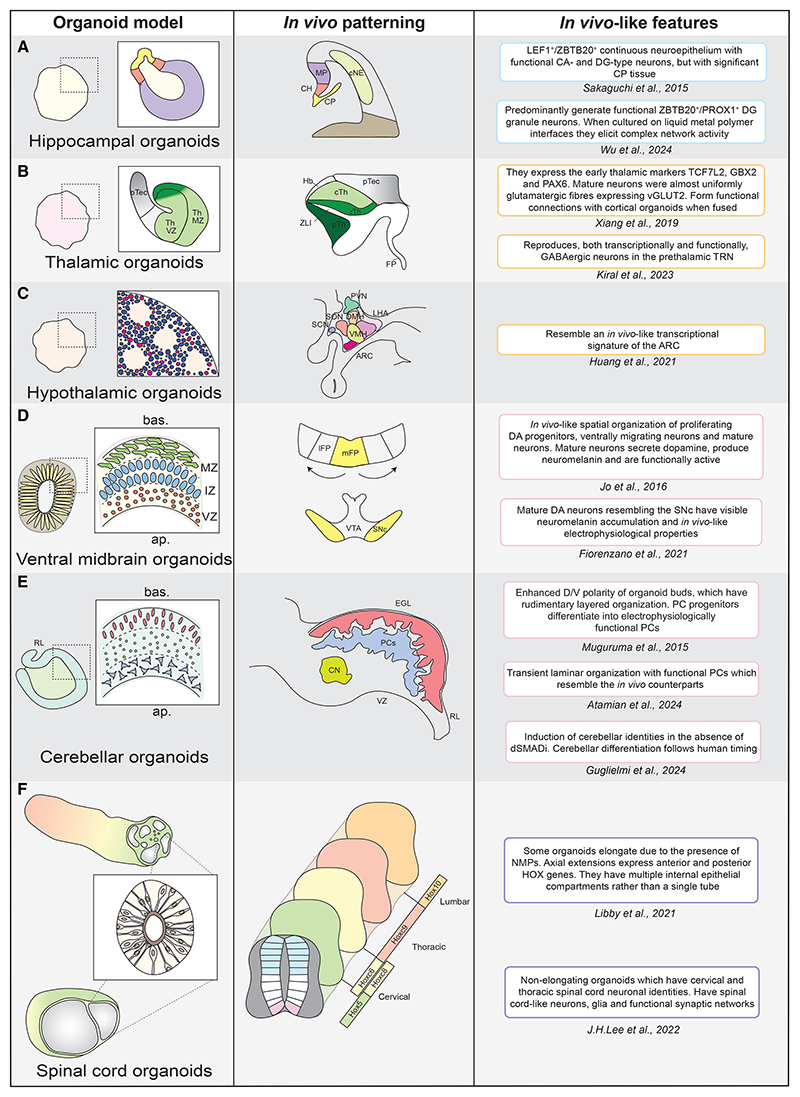
*In vitro* morphogenesis of regional brain organoids (A) Hippocampal organoids reproduce CA and DG identities. MP, medial pallium; CH, cortical hem; CP, choroid plexus; cNE, cortical neuroepithelium. (B) Thalamic organoids reproduce mainly caudal thalamic identities, which are also more abundant *in vivo*. Additional guidance by means of SHH signaling activation shifts identities anterior. Th, thalamus; VZ, ventricular zone; MZ, mantle zone; pTec, pretectum; rTh, rostral thalamus; cTh, caudal thalamus; ZLI, zona limitans intrathalamica; Hb, habenulae; FP, floor plate. (C) Hypothalamic organoids reproduce mainly ARC identities. PVN, paraventricular nucleus; LHA, lateral hypothalamic area; DMH, dorsomedial hypothalamic nucleus; SON, supraoptic nucleus; SCN, suprachiasmatic nucleus; VMH, ventromedial hypothalamic nucleus; ARC, arcuate nucleus. (D) Ventral midbrain organoids display *in vivo*-like apicobasal organization, and they predominantly specify into SNc identities. During development, SNc progenitors are born medially and then displaced laterally. As a consequence, the VTA develops medially with respect to the SNc. lFP, lateral floor plate; mFP, medial floor plate; VTA, ventral tegmental area; SNc, substantia nigra pars compacta. (E) Cerebellar organoids display robust induction of cerebellar cell types; in some instance those organize with a transient *in vivo*-like tissue architecture. PCs, Purkinje cells; EGL, external granule layer; CN, deep cerebellar nuclei; VZ, ventricular zone; RL, rhombic lip. (F) Spinal cord organoids reproduce aspects of spinal cord development. Based on the protocol, these organoids reproduce either cervical spinal cord identities or span across thoracic and lumbar domains.

*In vitro*, Sakaguchi et al. demonstrated that telencephalic organoids exposed to a pulse of CHIR99021 and BMP4 can mimic hem-derived signaling (Figure 1C).^64^ This treatment induces the formation of a continuous neuroepithelium, expressing LEF1 and ZBTB20, drivers of hippocampal competency. However, these organoids also displayed the presence of CP tissue, which is likewise hem derived.^64^ Long-term cultures of dissociated neurons from these organoids expressed CA and DG markers along-side mature morphologies.^64^ More recent protocol variations introduced WNT3A and purmorphamine (a SHH pathway activator) following CHIR99021 and BMP4 treatment^65^ (Figure 1C). Under these conditions, neuronal progenitors robustly acquired a DG granule neuron fate^65^ (Figure 2A).

Because D/V territories are interconnected, reproducing them within one organoid is essential for modeling telencephalic circuits. Although the application of recombinant ligands and small molecules at fixed concentrations can reliably bias differentiation, they often do so at the cost of cellular diversity, particularly within hippocampal and GE lineages, where temporal and spatial differences in morphogen levels are important. For instance, the levels of WNT activity act as a determinant between CA and DG identities, with CA specification requiring lower WNT signaling levels compared with DG.^63^ Similarly, within the GE, SHH signaling promotes MGE identity at the expense of CGE by preventing GSX2 expression.^66^

**Figure 3:**
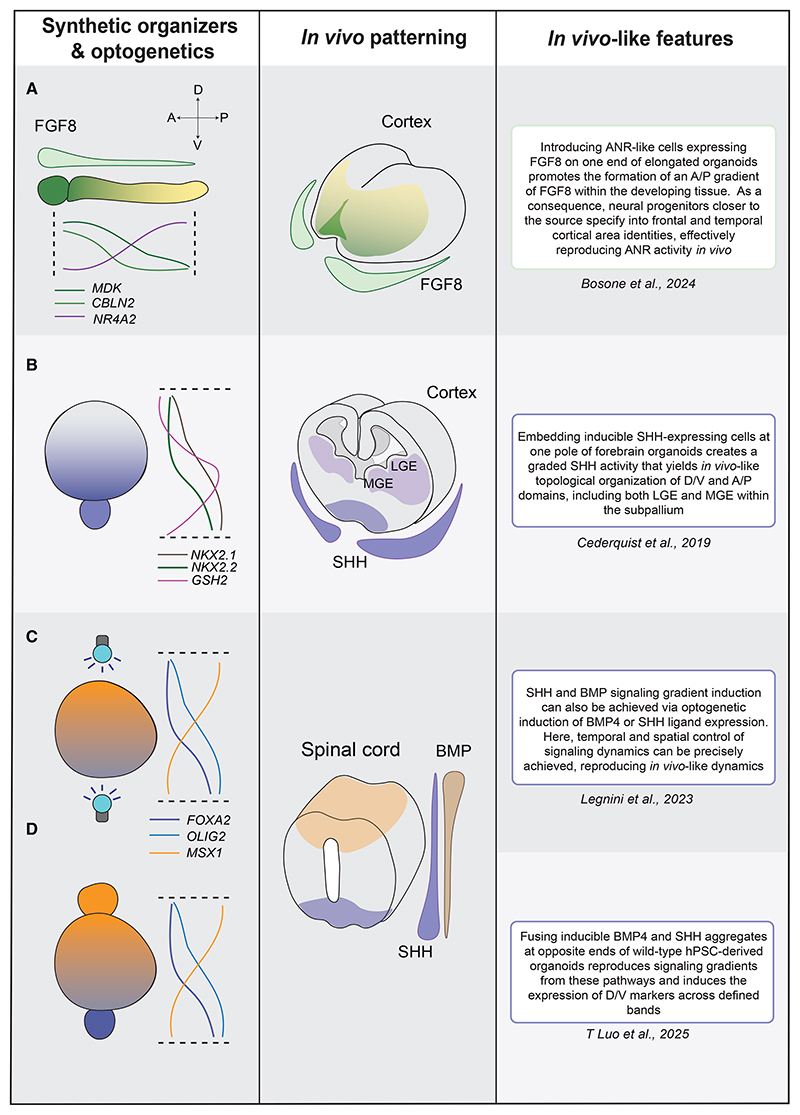
Reproducing signaling gradients *in vitro* with optogenetics and synthetic organizers (A) An ANR-like organizer combined with an elongated telencephalic organoid reproduces aspects of A/P patterning of the cerebral cortex, as shown by expression of anterior genes closer to the FGF8 source. (B) Inducible SHH-expressing cells on one pole of a telencephalic organoid promote the formation of different GE domains with *in vivo*-like topological organization. (C) Optogenetic activation of SHH and BMP signaling confers spatiotemporal control over signaling activity and the formation of signaling gradients within spinal cord organoids. Note that while optogenetic activation of either SHH or BMP has been described, simultaneous optogenetic activation within the same organoid, as shown here for illustrative purposes, is not yet technically possible. (D) Similar to (B), a synthetic organizer enables SHH and BMP signaling induction and gradient formation in spinal cord organoids. This results in the expression of different target genes at the expected distance from the source.

Notably, Bosone et al. introduced ANR-like cells expressing FGF8 on one end of elongated organoids, effectively creating an A/P gradient of FGF8 within the developing tissue^67^ (Figure 3A). This strategy promoted the specification of frontal and temporal cortical area identities closer to the signaling source^67^ (Figure 3A). Likewise, Cederquist et al. embedded inducible SHH-expressing cells at one pole of forebrain organoids, creating graded SHH activity that yielded an *in vivo*-like topological organization of D/V and A/P domains, including both LGE and MGE within the subpallium^68^ (Figure 3B). Therefore, reproducing spatially distinct sources of FGF, WNT, and SHH within the same organoid, in combination with the intrinsic self-organizing capacity of unguided telencephalic organoids, may enhance morphogenetic fidelity, providing a more faithful platform for studying human telencephalic development and disease.

### ZLI

The ZLI is a transverse signaling center located at the boundary between the thalamus and prethalamus in the developing diencephalon.^69^ It secretes SHH to pattern the prethalamus, thalamus, and hypothalamus along the D/V and A/P axes.^46^ FGF and WNT signaling further contribute to establishing diencephalic domains.^70^ Because the thalamus mediates corticospinal communication and sensory relay, significant efforts have been devoted to modeling it *in vitro*.

Shiraishi et al. developed one of the earliest thalamic models using mouse embryonic stem cells (mESCs).^71^ This early protocol relied on applying minimal guidance to posteriorize telencephalic aggregates using insulin, in combination with a MEK/extracellular signal-regulated kinase (ERK) inhibitor to suppress FGF signaling (as insulin can activate FGF signaling) and prevent specification of mesencephalic identities. BMP7 was also included, as its dorsalizing activity likely prevents ventral hypothalamic fates.

These conditions yielded apicobasally organized progenitors resembling the thalamic primordium, including Gbx2^+^/Tcf7l2^+^ caudomedial and Sox2^+^/Tcf7l2^+^ rostral populations (Figure 2B). Reproducing both domains in one model is key to studying disorders spanning this developmental boundary, such as epilepsy and neurocognitive syndromes.

Here, dependence on SHH signaling has been exploited to navigate across diencephalic domains. Indeed, regions closer to the SHH source (i.e., adjacent to the ZLI) are exposed to higher SHH concentrations and give rise to the thalamic reticular nucleus (TRN) and the rostral thalamus. In contrast, more caudal thalamic regions, experiencing lower SHH levels, specify into caudal thalamus.^72^ These rostrocaudal subdivisions are also distinguished by their neurotransmitter identity: the TRN and rostral thalamus are predominantly composed of γ-aminobutyric acid (GABAergic) neurons, whereas the caudal thalamus is enriched with excitatory neurons.^73,74^

Consistent with these *in vivo* observations, addition of SAG, a SHH signaling activator, to thalamic mouse organoids strongly increased the proportion of rostral progenitors.^71^ Similar results were obtained using hESC-derived thalamic organoids^75^ (Figure 1D), which, as mouse organoids, formed thalamic-like territories enriched in glutamatergic neurons in the absence of SHH. Upon addition of recombinant SHH, human thalamic organoids shifted toward rostral and ventral fates (Figures 1D and 2B), resembling the TRN.^76^

As both the thalamus and the hypothalamus depend on SHH signaling, independent specification of these two identities *in vitro* may not be trivial. Although thalamic organoids employ activation of SHH alongside BMP7 to bias toward medial diencephalic identities, to move further ventral, Huang et al. activated SHH signaling using combined purmorphamine, recombinant SHH, and SAG.^77^ This activation was initiated from day 0 of differentiation in the absence of BMP signals (instead of day 14 as for thalamic organoids^76^). WNT inhibition was also employed to prevent the induction of dorsalizing cues (Figure 1E).^77^

This protocol resulted in organoids transcriptionally resembling the human neonatal arcuate nucleus (ARC) (Figure 2C).^77^ Other hypothalamic domains, such as the lateral hypothalamus, ventromedial hypothalamus, and suprachiasmatic nucleus, were not observed. Whether this limited diversity may stem from the guided nature of this protocol remains unclear, as SHH signaling from the ventral forebrain neuroepithelium, including the telencephalic floor plate, is important for the specification of anteriotuberal hypothalamic regions.^78^

Integrated models reproducing co-development of telencephalon and diencephalon may be required to capture the full spectrum of thalamic and hypothalamic diversity. Alternatively, graded SHH signaling could further refine patterning. Indeed, polarized SHH delivery, as shown by Cederquist et al., not only improved subpallial specification but also led to the emergence of anterior and ventroposterior hypothalamic nuclei.^68^

### MHB

The MHB separates the mesencephalon from the rhombencephalon and patterns key structures, such as the substantia nigra (SN) and the anterior hindbrain, the latter including rhombomere 1 and giving rise to the cerebellum.^79,80^ In the MHB, WNT and FGF signaling act synergistically on both sides of the boundary to maintain and refine regional identities.^47,81,82^ Disruption of either WNT or FGF signaling impairs development of midbrain and anterior hindbrain structures.^40,83^ The divergence between midbrain and hindbrain fates is modulated by pre-patterning events during gastrulation, when differential WNT signaling drives the segregation of the neural plate into spatially distinct territories, anteriorly marked by OTX2 and posteriorly by GBX2.^84–86^ The interface between these early domains sets the later positioning and function of the MHB.^86,87^

### MHB—Rostral identities

In the midbrain, anterior to the MHB, the SN and ventral tegmental area (VTA) are the major sources of dopaminergic (DA) neurons in the human brain.^88,89^ The VTA is positioned dorsomedially relative to the SN and contains a heterogeneous mix of DA, GABAergic, and glutamatergic neurons.^90^ The SN is further subdivided into the pars compacta (SNc), which is rich in DA neurons, and the pars reticulata, composed primarily of GABAergic projection neurons.^91,92^

In this setting, alongside WNT and FGF signals, SHH from the ventral neural tube is essential to establish DA competency.^93^ Medial DA progenitors are exposed to, and terminate, SHH signaling earlier than lateral progenitors. Consequently, medial progenitors preferentially generate KCNJ6-expressing DA neurons that populate the SN and VTA, whereas lateral progenitors give rise predominantly to CALB1-expressing neurons that localize to the ventromedial VTA.^94,95^

To reproduce ventral midbrain development *in vitro*, Jo et al. combined WNT, FGF8, and SHH activation to pattern hESCs^96^ (Figure 1F). Under these conditions, nascent organoids expressed DA progenitor markers and exhibited evidence of *in vivo*-like spatial organization, with proliferating progenitors, ventrally migrating neurons, and more mature neurons organizing within distinct layers (Figure 2D). Bulk RNA-seq analysis showed a transcriptional signature closely resembling that of the human prenatal midbrain and midbrain DA neurons.^96^ Using a similar approach, Fiorenzano et al. confirmed these findings and identified a predominant population of SNc-like DA neurons using scRNA-seq^97^ (Figures 1F and 2D). These DA neurons developed visible pigmentation from intra- and extracellular neuro-melanin accumulation, appearing as dark granular deposits, and exhibited functional electrophysiological properties resembling their *in vivo* counterparts.^96,97^

Given the involvement of SNc DA neurons in Parkinson’s disease, these models are potentially revolutionary for interrogating disease mechanisms. Improving the *in vitro* representation of VTA neuronal diversity would expand their utility to conditions affecting mesocorticolimbic circuits, such as schizophrenia, addiction, and depression. Because SHH exposure timing is a key determinant of SN versus VTA identity, adjusting the onset and duration of SHH signaling *in vitro* could enable the balanced emergence of both populations. Moreover, since levels of WNT signaling are important for VTA fate, physiological representation of WNT signaling levels could also promote co-emergence. Indeed, high WNT signaling activity participates in steering neural progenitors toward a VTA identity by upregulating OTX2 and suppressing SOX6, an SNc marker.^98,99^

In contrast, modulation of FGF signaling is important for establishing DA neuron identity along the A/P axis. In its absence, midbrain DA progenitors can be redirected toward diencephalic-like fates marked by Pou4f1 expression.^100^

### MHB—Caudal identities

On the posterior side of the MHB, a combination of WNT and FGF signals patterns the cerebellum. Although analogous instructive cues act on both sides of the boundary, a body of evidence suggests that FGF levels are important in establishing cerebellar competency. When beads containing recombinant FGF8 are implanted in the prospective caudal diencephalon or midbrain of chick embryos, the surrounding tissue develops into two ectopic, mirror-image midbrains. However, cells in direct contact with the bead (presumably exposed to higher FGF levels) develop toward isthmic nuclei and a cerebellum-like structure.^101^ Furthermore, disrupting or reducing Fgf8 signals at the MHB shifts cerebellar fate toward posterior midbrain identities,^102^ further suggesting that cerebellar specification relies on higher FGF signaling levels compared with the midbrain.^103^

*In vitro* generation of cerebellar organoids was built on the idea that, if an MHB-like state can be induced in at least a subset of progenitors, differentiation into cerebellar identities may occur without additional cues, effectively reproducing the main progenitor zones: the ventricular zone (VZ) and the rhombic lip (RL).^104^ *In vivo*, these regions give rise to distinct neuronal lineages. The VZ produces GABAergic neurons, including Purkinje cells (PCs), interneurons (such as basket, stellate, and Golgi cells), and some inhibitory neurons of the deep cerebellar nuclei. In contrast, the RL generates glutamatergic neurons, including granule cells (GCs), unipolar brush cells, and most of the excitatory neurons of the deep cerebellar nuclei.^84,105^

To reproduce cerebellar diversity, Muguruma et al. tested different posteriorization paradigms in mESCs. Addition of either Fgf8 or Wnt1 alone failed to induce expression of the cerebellar master regulator *En2*, which was instead achieved with a combination of Fgf2 and insulin. Treated aggregates went on to express endogenous *Wnt1* and *Fgf8*, suggesting that a self-sustaining MHB-like signaling center had been established.^104^ The superior EN2-inducing capacity of FGF2 compared with FGF8 *in vitro* was also shown in 2D settings.^106^

This posteriorization strategy was later applied to hiPSC-derived brain organoids, leading to similar formation of VZ and RL-like domains and the emergence of PCs^107^ (Figure 1G). Since then, the combination of insulin and FGF2 has been extensively used to induce cerebellar identities. However, increasing evidence suggests that additional cues may be required. Under conditions in which FGF signaling is activated in combination with insulin,^108,109^ organoids often retain a significant proportion of forebrain identities. These are fully suppressed when high doses of FGF2,^109^ currently available as a preprint and not yet peer reviewed, or FGF8,^108^ are accompanied by WNT signaling activation using CHIR99021 (Figure 1G).

Furthermore, following FGF2 or FGF8 exposure, addition of another FGF ligand, FGF19 (the human ortholog of mouse Fgf15) enhances D/V polarity within organoid buds.^107^ When combined with SD1, a CXCR4 ligand involved in GC progenitor migration, cerebellar organoids developed a partially layered organization, with PC progenitors positioned in between the VZ and an RL-derivative zone,^107^ albeit such organization is only transient^108^ (Figure 2E). Under these conditions, developing PCs became functional,^108^ albeit lacking the typical complex dendritic arborization. Such characteristics were only observed when PCs were co-cultured with mouse cells derived from the embryonic RL.^107^ Similarly, GC progenitors extended axons and developed the characteristic T-shaped morphology when co-cultured with mouse GCs.^107^

Unlike other regional brain models, current cerebellar organoids successfully recapitulate much of the cellular diversity observed in the developing human cerebellum.^108^ However, this is not yet followed by tissue-scale organization of PCs and GCs, characteristic of *in vivo* development, which is important for modeling cerebellar function *in vitro*.

Remarkably, compared with anterior brain organoid models, posterior organoids often display smaller buds.^109,110^ This difference may reflect intrinsic morphogenetic properties of the MHB, which *in vivo* forms a pronounced constriction of the neural tube.^79^ Evidence from developmental models suggests that high FGF signaling can promote actomyosin-mediated cell contraction, potentially contributing to this morphological feature.^111,112^ Although such constriction is thought to help position and maintain the MHB signaling center, *in vitro* it may come at the cost of reducing the surface area available for establishing robust D/V and A/P patterning. In human development, scaling of the cerebellum requires extensive surface expansion to accommodate RL- and VZ-derived neurons, a process that may be partially uncoupled from MHB constriction. Therefore, understanding whether compact bud morphologies are an unavoidable consequence of FGF signaling activation *in vitro* or whether they can be modulated without loss of cerebellar identity will be essential for modeling cerebellar morphogenesis *in vitro*.

### Tailbud and caudal organizer-like activity

During late gastrulation and axis elongation, the tailbud region coordinates formation of the posterior body axis.^48^ It gives rise to two types of progenitors: neuromesodermal progenitors (NMPs), expressing TBXT and SOX2, which give rise to spinal cord neurons and somites, and axial progenitors that give rise to the notochord.^113–115^ As the body axis extends, the notochord forms along the midline, somites bud off the paraxial mesoderm laterally, and the neural tube forms dorsally.^116^ These events are orchestrated by intersecting gradients of WNT, FGF, BMP, and RA signaling within the tailbud environment. Namely, BMP signaling supports correct notochord and floor plate formation, while low levels promote paraxial mesoderm and neural fate.^117–119^ WNT and FGF signaling contribute to maintaining NMPs, supporting both continued axial elongation and the sequential production of neural and mesodermal derivatives.^120^ As elongation proceeds, RA signaling promotes neural differentiation and A/P patterning by counteracting WNT and FGF signals and restricting the NMP domain.^113,121^

The development of the notochord and spinal cord is closely linked.^122,123^ The notochord and the floor plate secrete SHH, which contributes to the establishment of ventral spinal cord identities such as motor neurons.^124^ As such, the integration of these different signaling inputs is important for the specification of spinal cord cell types.^125^

In line with these notions, spinal cord organoids typically employ posteriorization prior to neural commitment. For instance, Libby et al.^126^ cultured human pluripotent stem cells (hPSCs) in mTeSR1 medium, which contains both FGF and activin (TGF-β) to promote a pluripotency state, and caudalized them with CHIR99021 2 days before neural differentiation and aggregation (Figure 1H). Following aggregation, organoids were treated with dSMADi, RA, and purmorphamine to promote neural tube specification.^126^ Some organoids developed singular axial extensions, which correlated with the presence of NMPs, suggesting the emergence of a tailbud organizer-like region and a rudimentary TBXT^+^ longitudinal structure resembling a notochord. Furthermore, elongated organoids displayed expression of anterior and posterior HOX genes, showing that A/P patterning of the spinal cord could be reproduced (Figure 2F). However, similar to anterior brain organoids, they often displayed multiple internal epithelial compartments rather than forming a single cohesive neural tube.^126^

More recent protocols have employed similar pre-patterning strategies. For instance, Lee et al. used a combination of CHIR99021 and the TGF-β signaling inhibitor SB-431542 for 3 days to induce posteriorization prior to aggregation^127^ (Figure 1H). This step was followed by treatment with FGF2, which led to the transient emergence of NMPs in the absence of TBX6 expression, suggesting acquisition of neural, rather than mesodermal identities. 4 days after aggregation, FGF2 was replaced with RA to promote spinal cord specification. Importantly, elongation was not reported under these settings, and spinal neuronal identities were restricted to cervical and thoracic spinal cord. Nevertheless, these organoids developed spinal cord-like neurons, glial cells, and functional synaptic networks.^127^

D/V distribution of spinal cord neuronal types relies on opposing SHH and BMP signaling gradients from the floor plate and roof plate, respectively. Ventral neurons develop into motor neurons and ventral interneurons, whereas the dorsal region specifies into sensory interneurons. Consequently, spatial and temporal control of these signaling pathways *in vitro* is sought for appropriate reproduction of spinal cord identities. Legnini et al. engineered an optogenetic system to induce SHH or BMP4 ligand expression in caudalized neural organoids.^128^ This system utilizes a split Cre recombinase that is reconstituted upon light stimulation. Once active, Cre mediates a switch from red fluorescent protein to mNeon expression, concomitantly driving the expression of the ligand of interest. Using this system, they illuminated only one pole of the organoid and achieved graded expression of SHH target genes, such as *FOXA2, OLIG2*, and *NKX6-1*, recapitulating the distance of their expression domains from the SHH source. Similar results were acquired with a photoinducible BMP4 line, which led to induction of dorsal markers such as *MSX1*^128^ (Figure 3C).

Similarly, Luo et al. generated two hPSCs lines expressing BMP4 (iBMP4) and SHH (iSHH), respectively.^129^ They fused iBMP4 and iSHH aggregates at opposite ends of wild-type hPSC-derived organoids, which were first treated with dSMADi to promote neural fate, followed by CHIR99021 and RA to promote caudalization. Upon doxycycline induction, the organoids exhibited expression of D/V markers across defined bands, closely mimicking the spatial organization of neural progenitors across the embryonic neural tube (Figure 3D). In terms of A/P patterning, the organoids were generally positive for *HOXB4*, a marker of the posterior hindbrain and anterior spinal cord. These neural tube organoids closely resemble the human embryonic spinal cord at Carnegie stage 12 and show higher transcriptomic similarity to human rather than mouse neural tube tissue.^129^

## Integrated Patterning Of The Human Neuraxis

Modeling different organizers in the brain opens up the possibility of recapitulating the development of specific brain regions *in vitro*. These approaches have been particularly valuable for studying biological processes and diseases that affect defined brain areas. However, as brain domains are developmentally coupled and interconnected, the need to reproduce their coordinated development *in vitro* is self-evident. One of the most tractable strategies employed so far makes use of assembloids, in which region-specific organoids are combined after their A/P and D/V identities have been established, allowing them to interact in ways that mimic *in vivo* spatial relationships. Other approaches are aimed at generating synthetic neural tubes or brain-body gastruloids, in which organizers, morphogen gradients and tissue geometry are established from the outset, allowing A/P and D/V patterning to emerge in a coordinated, embryo-like fashion. In this way, they bring *in vitro* modeling closer to the developmental logic of the human CNS, with the potential to reveal how regional identities, boundaries, and connectivity co-emerge along the entire human neuraxis.

### Assembly of regional organoids

Provided that the morphogens driving brain differentiation toward a given identity *in vivo* can be discerned from the literature and applied to organoids, assembloids open up avenues for the study of interactions between different brain regions in any pertinent combination.

This plug-and-play approach to studying regional interactions in the brain must forfeit some of the nuance of regionalization and patterning seen during *in vivo* development. It likely also affects signaling gradients, intermediary areas between core regional identities, and natural boundary establishment. Even so, the ability to define the identities of constituent organoids in a reproducible way may make assembloids a tractable system for probing appropriate questions of regional interactions.

**Figure 4:**
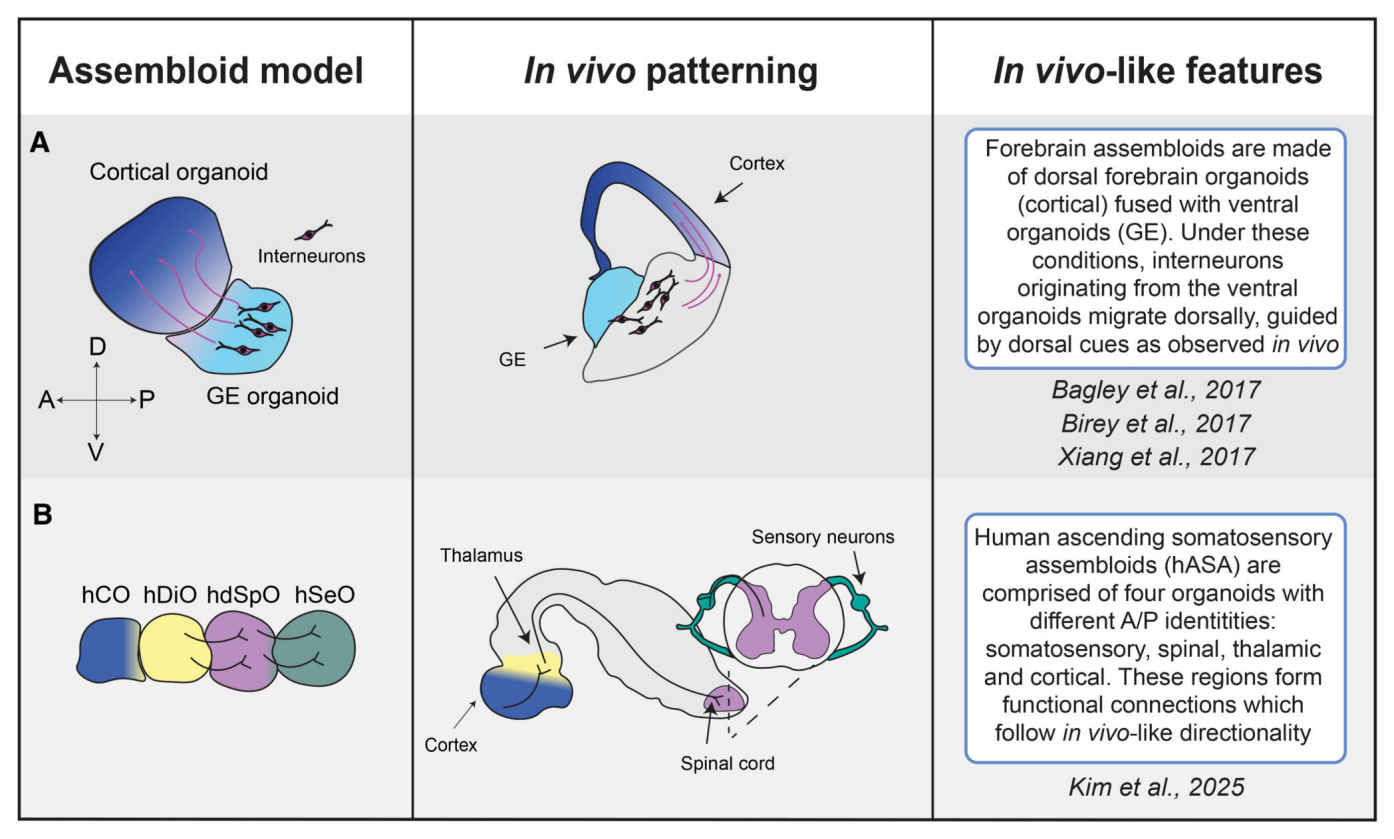
Combining regional organoids into assembloids (A) Fusing cortical and GE organoids recapitulates dorsal migration of interneurons within the human telencephalon. GE, ganglionic eminence. (B) The most complex assembloid containing the following: hCO, human cortical organoid; hDiO, human diencephalic organoid; hdSpO, human dorsal spinal cord organoid; hSeO, human sensory neuron organoid. This model recapitulates aspects of the human ascending somatosensory system.

For example, assembloids have been used to recreate morphogenetic interactions across telencephalic domains, including interneuron migration from ventral to dorsal forebrain. Fusion of ventral forebrain organoids with dorsal cortical organoids recapitulated the directed migration of interneurons seen *in vivo*-^56,58,59^ (Figure 4A). This paradigm has since been applied to model neurodevelopmental disorders, such as Timothy syndrome, where interneurons derived from patient hiPSCs displayed reduced migration speed and shorter migratory steps, linked to defects in myosin phosphorylation and altered GABA sensitivity.^56,130^ Thus, assembloids provide windows to study phenomena of biological development and disease across brain regions.

Prior to refinement of protocols for deriving regional brain organoids, attempts to establish cortico-spinal-muscle circuitry involved combining cortical brain organoids with mouse spinal cord explants. This system was able to induce the paraspinal muscle tissue to contract.^27^ With subsequent successful patterning of spinal cord organoids and skeletal muscle organoids, all three components of the cortico-spinal-muscle circuitry were able to be patterned. These three-organoid assembloids model that entire circuit in wholly human stem-cell-derived tissue.^131^

Patterning organoids and combining them into assembloids allows to probe connectivity specific to brain regions implicated in diseases. Examples include *ARID1B* mutations in corpus callosal connectivity, Huntington’s defects in striato-nigral connections, and Phelan-McDermid syndrome phenotypes in cortico-striatal projections.^132–134^ Interestingly, cortico-striatal projections, but not reciprocal projections, were observed in cortico-striatal assembloids, reminiscent of the *in vivo* anatomical arrangement. Therefore, modular patterning using assembloids may not just generate random connections by virtue of having neurons. Under the right circumstances, these models may be anatomically or physiologically faithful as well. Perhaps the most advanced model among these is the human ascending somatosensory assembloid, a four-part assembloid that integrates somatosensory, spinal, thalamic, and cortical organoids to model the spinothalamic pathway^135^ (Figure 4B).

Intriguing studies are seeking to retain some of the nuance to developmental patterning that was initially lost with the driving of spheroids to uniform identities for use in assembloids. A similar approach integrating a spheroid, designated as an organizer, into an assembloid to influence patterning of the other constituent organoid, now allows to study the effect of the floor plate organizer on axon guidance.^136^ These approaches may once again highlight the importance of signaling gradients and interactions in patterning these *in vitro* models.

### Patterning of synthetic neural tubes

CNS organoids and assembloids recapitulate much of the cell-type diversity and spatiotemporal patterning within individual regions of the brain and spinal cord. However, due to the uniform distribution of morphogens in culture and self-patterning, organoids exhibit a high degree of variation in their structure, yield anatomically incorrect features, and can induce poor cell fate patterns.^137,138^ This commonly leads to multiple developing neuroepithelial rosettes and ventricular lumens within the same organoid. Furthermore, the nature of organoid self-organization leads to hypoxic regions and necrotic cores, disrupting the profile and response to morphogens.^139^ To overcome these limitations and better recapitulate tissue-scale geometry, bioengineering and micropatterning strategies have been developed to generate synthetic neural tubes *in vitro*. These approaches can be coupled with microfluidic devices to establish organizer regions and stable morphogen gradients.^137^

**Figure 5:**
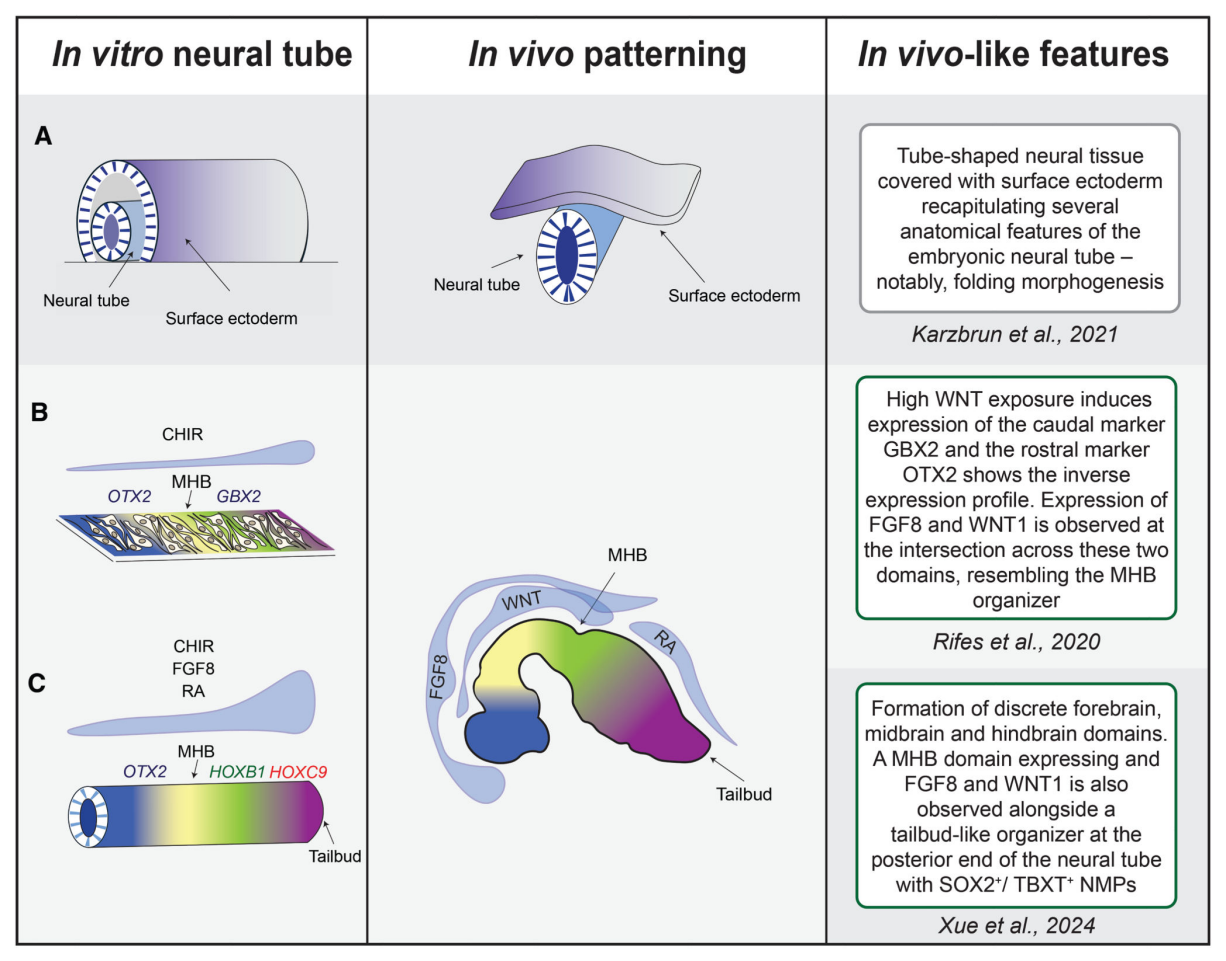
Synthetic neural tube and microfluidics to reproduce A/P patterning (A) Synthetic neural tube surrounded by surface ectoderm. After initial 2D micropatterning, folding morphogenesis occurs because of self-organization. (B) Formation of an ectopic WNT signaling gradient using a microfluidic device. Under these conditions, cells exposed to high WNT levels within the micropattern express GBX2, conversely the ones exposed to lower levels express OTX2. (C) An ectopic gradient of WNT, FGF, and RA further refines A/P patterning into forebrain, midbrain, hindbrain, and a tailbud-like organizer.

Using surface micropatterning of stem cells, Karzbrun et al. created a controlled 2D array of PSCs with defined size and density.^138^ Embedding these arrays in Matrigel enabled a transition to a 3D pluripotent epithelium that wrapped around a single lumen. Neural induction with SB-431542 followed by BMP4 exposure triggered self-organized pattern formation and folding morphogenesis, yielding a tube-shaped neural tissue covered with surface ectoderm and recapitulating several anatomical features of the embryonic neural tube^138^ (Figure 5A).

Rifes et al. instead seeded a confluent monolayer of cells along a longitudinal Matrigel substrate within a microfluidic device.^140^ This system enabled the formation of a precisely defined WNT rostrocaudal concentration gradient (0–2 μM CHIR99021) mimicking A/P patterning.^140^ Application of such a gradient, using neural induction medium with dSMADi, induced expression of the caudal marker *GBX2* at high CHIR99021 exposures, whereas *OTX2*, a rostral marker, showed the inverse expression profile^140^ (Figure 5B). Notably, similar to *in vivo* development, expression of *FGF8* and *WNT1* was observed at the intersection across these two domains, resembling the MHB organizer. Furthermore, floor plate identities could be induced upon activation of SHH signaling pathway.^140^

More recently, Xue et al. employed microcontact printing to form an array of Geltrex adhesive islands and guide stem cell organization into a continuous rostrocaudal neural tube or discrete region-specific domains.^141^ Similarly to Karzbrun et al., a ventricular lumen was identified along the length of the synthetic neural tube.^141^ Using a microfluidic device, the authors established rostrocaudal gradients of WNT activity (also using CHIR99021), FGF8, and RA, which patterned the neural tube into discrete forebrain, midbrain, and hindbrain domains (Figure 5C). Strikingly, they observed the formation of a MHB domain expressing *WNT1* and *FGF8*, as well as a tailbud-like organizer at the posterior end of the neural tube with SOX2^+^/TBXT^+^ NMPs that were able to differentiate into presomitic mesoderm cells or motor neuron progenitors.^141^

The combination of synthetic neural tube models with microfluidic devices represents a powerful platform, enabling the emergence of key developmental features, such as MHB formation, floor plate induction, and axial progenitor specification, with high spatial fidelity. However, long-term culture of complex neural tissues typically relies on dynamic systems, such as orbital shakers, perfusion bioreactors, organotypic slice cultures, or vascularization-based approaches. All these methods improve oxygenation, viability, and maturation, but are not easily compatible with fixed microfluidic configurations. Integrating fine-tuned morphogen delivery with culture formats that sustain growth over weeks to months will therefore require novel bioengineering solutions that merge gradient-generating devices with scalable, perfused, and mechanically supportive culture systems. Achieving this integration will be essential for harnessing the developmental precision of synthetic neural tubes, while maintaining the long-term viability and functional maturation necessary to model human CNS development and disease.

### Brain-body gastruloids

During gastrulation, the emergence of the primitive streak at the posterior end of the embryo lays down the emergence of the A/P axis. This step is followed by the specification of the three primary germ layers and the establishment of the entire body plan.^142,143^ Primitive streak formation is induced in the posterior region of the epiblast by WNT and Nodal signaling pathways, which drive the generation of primitive streak-derived mesendoderm.^144–146^ Within the anterior epiblast, inhibition of WNT and NODAL signaling protects rostral regions from posteriorization and allows the ectoderm to differentiate into neuroectoderm and eventually, the CNS.^145,147,148^ Brain organoids provide a useful tool to isolate neurodevelopmental mechanisms, but they model different regions of the brain in isolation, neglecting the coordinated development at the base of brain ontogeny. Therefore, reproducing human gastrulation *in vitro* could, in principle, reproduce the development of anterior and posterior brain domains, maintaining their relationships intact.

**Figure 6:**
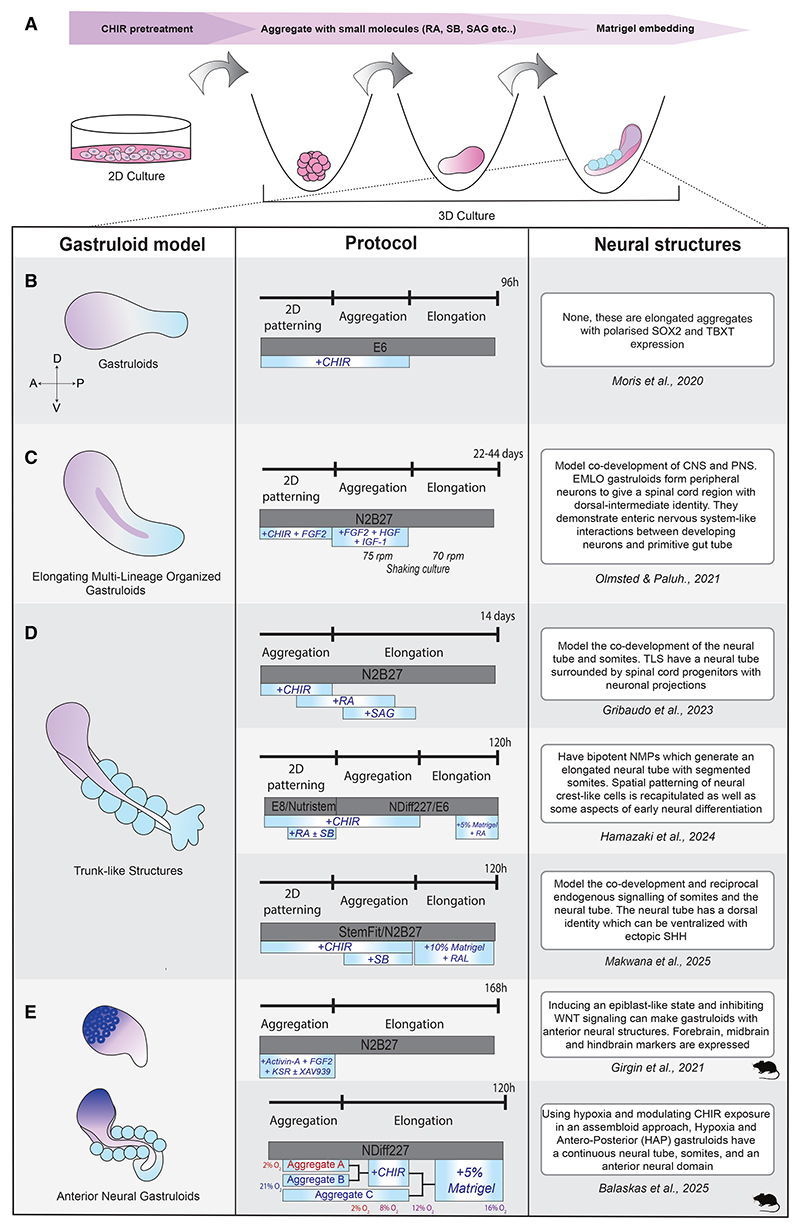
Coordinated CNS patterning using brain-body gastruloids (A) Schematics representing the general steps in the gastruloid protocol. (B) Protocol for generating human gastruloids. These do not typically display anterior neural structures. (C) EMLO gastruloids display the formation of a partial spinal cord structure and an enteric nervous system. (D) Protocols for obtaining trunk-like structures. These gastruloids form somites and the neural tube; however, anterior neural tissue is normally not detected. (E) Anterior neural gastruloids recapitulate anterior and posterior neural patterning; however, anterior neural tissue is normally poorly organized.

A holistic approach to modeling early stages of axial specification was introduced when van den Brink et al. showed that mESCs can spontaneously break symmetry *in vitro*, establish an axis, and specify germ layers in a manner reminiscent of gastrulation.^149^ This phenomenon was later confirmed in hESCs, generating the first human 3D model of gastrulation, or “gastruloid.”^150^ In their simplest form, gastruloids are stem cell aggregates that, upon exposure to CHIR99021, elongate along an A/P axis (Figure 6A). More recent protocols use elaborate cocktails of morphogens to achieve neural tube-like structures, organized somite pairs, and, in some cases, primitive endoderm-derived structures,^151–157^ with the most complex human gastruloid model matching a Carnegie stage 13 embryo.^153^

Current gastruloid models can be broadly grouped into four categories: gastruloids, trunk-like structures (TLSs), elongating multi-lineage organized (EMLO) gastruloids, and anterior neural gastruloids (Figures 6B–6E). Traditional gastruloids are teardrop-shaped aggregates with a SOX2^+^/TBXT^+^ region at one end^150,154^ (Figure 6B). Although these models are useful for understanding individual morphogen contributions toward axis formation and initial germ layer specification, they do not fully capture the morphological complexity of *in vivo* gastrulating embryos. More sophisticated models, such as the TLS, with a “trunk” and a SOX1^+^/PAX6^+^ neural tube-like structure flanked by paired epithelialized somites (Figure 6D), have since been adopted.^156,157^

*In vivo*, neural tube development, somite formation, and apicobasal polarity rely upon the extracellular matrix protein, fibronectin, which can be mimicked by embedding gastruloids in Matrigel.^156,158,159^ Further morphological complexity is achieved through additional small-molecule interference. Application of RA has been suggested to overcome limitations of previous gastruloid models by enabling coordinated development of neural tube-like and paraxial mesodermal structures.^153^ Addition of SHH agonists provides floor plate-like signals, normally coming from the notochord, ventralizing somites and the neural tube-like structure.^157^

To date, TLSs have had limited utility for interrogating neural development in humans. Pioneered as an *in vitro* model of central and peripheral nervous system co-development, EMLO gastruloids offer an alternative route for investigating early organogenesis and multi-tissue interactions^155^ (Figure 6C). Yet, although gastruloids can provide a means to remarkably reproduce either central and/or peripheral neuronal systems, a human gastruloid model with neural cell types located more anterior to the midbrain is still lacking. Since the emergence of gastruloid protocols is relatively recent compared with cerebral organoid protocols, they are not without teething issues.^20^ Indeed, there is still variation both within and between batches in terms of overall morphology, neural tube length, and somite organization.^160,161^ Poor protocol reproducibility and variability issues aside, creating anterior neural tissue within gastruloids remains a challenge.

A likely explanation for the lack of anterior neural tissue is the inability to reproduce spatial differences in WNT levels within the epiblast. Instead, gastruloid protocols employ homogeneous WNT activation across all cells through the application of fixed concentrations of CHIR99021. How the human anterior hypoblast is protected from posteriorizing morphogens is unclear and is much better described in other species. For instance, in mice, the anterior visceral endoderm (AVE) acts analogously to the human anterior hypoblast.^162,163^ There, AVE positioning and emergence are constrained by signals from the extraembryonic ectoderm, and its formation is initiated through Nodal signaling between the epiblast and visceral endoderm.^147,164–166^ Although mouse gastruloid models are similarly devoid of anterior neural tissue, Girgin et al. were able to generate an elongated gastruloid with fore-brain-, midbrain-, and hindbrain-like tissues^167^ (Figure 6E). This result was achieved through induction of epiblast identity by treatment of mESCs with FGF2 and activin A, alongside inhibition of WNT signaling through XAV939. In this case, XAV939 presumably acts similarly to the AVE by protecting against posteriorizing WNT signals and preventing premature mesodermal differentiation, as observed in traditional gastruloid models. This protocol is a prime example of how understanding *in vivo* gastrulation processes can be utilized to guide *in vitro* models.

Along these lines, and building upon initial findings that brain-like tissue can be generated in gastruloids exposed to a combination of CHIR99021 and hypoxia (2% oxygen),^168^ Balaskas et al., developed hypoxia anteroposterior (HAP) gastruloids,^169^ a study currently available as a preprint and not yet peer reviewed. Fusing CHIR99021- and hypoxia-exposed cells with cells cultured in normoxia (21% oxygen) generates HAP-gastruloids with spinal cord, somites, and some endoderm-derived structures. Strikingly, unlike traditional models, they also contain OTX2^+^ anterior brain-like tissues^169^ (Figure 6E). Therefore, understanding how the human anterior epiblast is shielded from WNT exposure will inform future gastruloid protocols to achieve an *in vivo-*like balance between WNT agonism and antagonism to initiate the coordinated emergence of anterior and posterior neural tissues.

## Outlook and Future Directions

The various approaches outlined in this review highlight the remarkable progress made in recapitulating human neural development *in vitro*. This advance is particularly striking when considering neural diversity in terms of transcriptional states. Although morphological fidelity across posterior brain regions and spinal cord does not yet match *in vivo* development, there are encouraging signs that this gap may soon be closed.

One emerging observation is that strong ectopic guidance can increase the reproducibility of specific cell types but may do so at the cost of *in vivo* patterning and morphogenetic complexity. In contrast, strategies that promote organizer-like morphogen gradients, whether in regionalized organoids or synthetic neural tube systems, tend to enhance neural diversity and spatial patterning, highlighting the importance of reproducing appropriate signaling dynamics *in vitro*. While optogenetics and synthetic organizers were mainly applied in patterning spinal cord organoids, these technologies could help stabilize relationships between dorsal-medial pallium and ganglionic eminence identities in telencephalic organoids, SHH-mediated rostrocaudal diversity in the thalamus, WNT and FGF signaling gradients at the MHB, or early WNT signaling differences in the epiblast.

Reproducing neuronal relationships within and across CNS regions is important for advancing our understanding of development and disease. The transition from 2D to 3D systems has enabled complex features rarely seen in traditional cultures, such as the emergence of synaptic connectivity. Yet, heavily guided protocols often compress neural identities to a limited set of cell types, likely due to the uniform application of the different ligands/small molecules. This necessarily limits the meaningful emergence of an *in vivo*-like network. For example, protocols that lack either GABAergic or glutamatergic neuron counterparts, which locally buffer and refine circuit outputs, can undermine physiological network function. This outcome is particularly relevant for disease modeling, where partial recapitulation of network architecture may obscure or exaggerate disease phenotypes compared with the *in vivo* patient context.

Although reproducing organizer signals is central to shaping morphogenesis, additional considerations are essential. Increasing evidence suggests that signaling gradients do not always conform to a strictly deterministic model in which specific levels invariably yield defined cell fates.^170–172^ Instead, aspects of cell specification are likely influenced by stochastic processes, meaning that gradient formation *in vitro* should aim to mirror *in vivo* conditions reproducing spatial profiles and steepness, rather than engineering “dose-fate” relationships. Moreover, developmental patterning occurs within a continuous dynamic context, in which changing environmental cues actively refine cell behavior in real time, ensuring both robustness and adaptability in tissue formation.^170,173^

A key role for self-organization is clearly illustrated by early organoid models, in which neuronal progenitors assemble into cytoarchitectures that are strikingly similar to the *in vivo* cortical plate. However, this structural fidelity tends to diminish as models move beyond the cortex, unless the spontaneous emergence of organizer-like structures takes place, as observed in some unguided telencephalic differentiations.^26^ Although these outcomes are highly variable, stabilizing such organizers and their associated gradients using the technologies discussed above may help strike an optimal balance between self-organization and guided differentiation, channeling morphogenesis toward more reproducible and physiologically relevant outcomes.

Certain aspects of neural development may also require influences that are difficult to reproduce *in vitro*, such as those provided by extraembryonic tissues, which is particularly relevant for gastruloid models. For instance, it has been shown that mESCs, when mixed with trophoblast stem cells and extraembryonic endoderm stem cells, can self-organize into embryoids capable of recapitulating key neurulation hallmarks, such as head-fold morphogenesis.^174^ This result shows how employing the critical, yet unclear, influence of extraembryonic tissues can recapitulate neurodevelopmental events without the need for external small-molecule interference. Similarly, refined development of germ layer specification in a gastruloid context could enable the study of interactions between neuronal and non-neuronal tissues, such as vasculature, which is fundamental in sustaining and refining brain morphogenesis.

The culture environment further shapes morphogenetic outcomes, as illustrated by comparisons between conventional multi-bud organoids and synthetic neural tubes. While formation of an individual neural tube is preferable to model the *in vivo* counterpart, it will be exciting to test whether, and for how long, these models can support regional morphogenesis without extrinsic support. Similar to the use of Matrigel in gastruloids, extracellular matrix scaffolds may help in this regard. Long-term maturation, however, remains a challenge also for gastruloid systems, which typically do not progress beyond 2 weeks in culture. Although these models may more closely capture early *in vivo* developmental events, regional organoids have proven far more robust in long-term culture, particularly under air-liquid interface conditions.^27^ They may therefore remain the most practical and informative model for investigating later stages of neuronal maturation.

Overall, the field is approaching these challenges from multiple angles. Progress will likely come from intersecting these domains, combining strategies for gradient formation, self-organization, extrinsic tissue influence, and optimized culture systems to improve morphological fidelity and bridge the gap between fate and form in ways matching the embryo.
